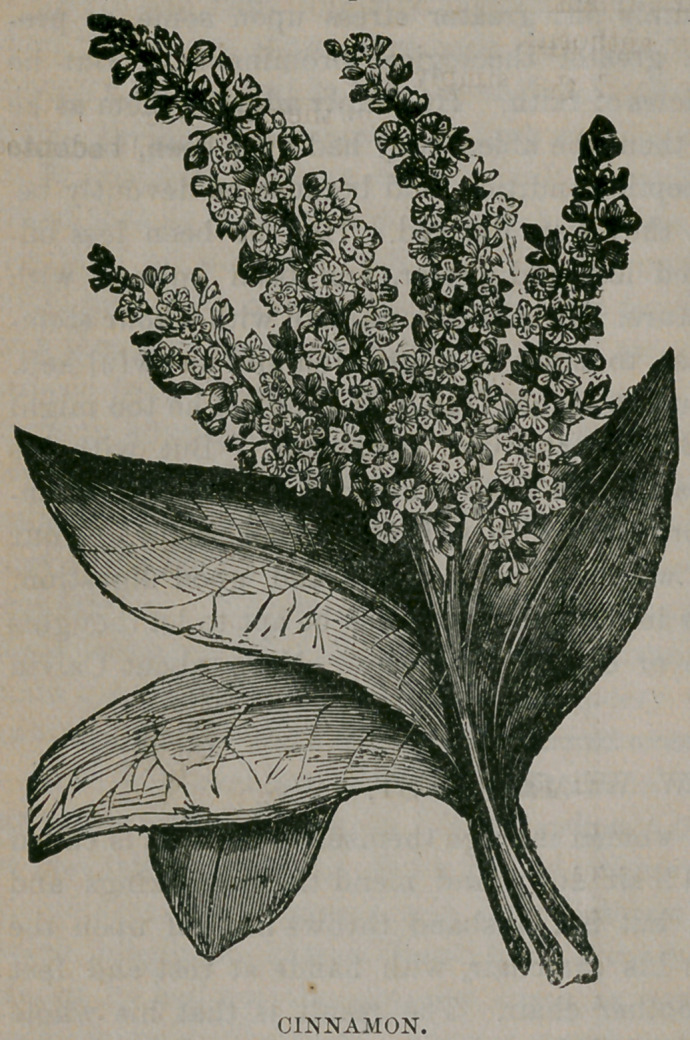# Cinnamon

**Published:** 1889-05

**Authors:** 


### CINNAMON.

Cinnamon is the bark of a small tree, the *Cinnamonum Zeylanicum*, which, as its name imports, is a native of Ceylon, and chiefly cultivated there, though it is raised also in Java. The tree is very graceful; the leaves, which are red in spring, become thick, leathery, and glossy-green as the summer advances; they are netted with raised veins on the under side, and are placed opposite each other on the stem; the flowers are greenish-white, and grow in small, loose clusters at the termination of the branches. The trees require a rich, light soil, and also shade; they are, therefore, planted in open glades of the forest, where a few large timber trees remain to shelter them; this greatly contributes to the beauty of the cinnamon harvest, when the natives assemble to strip the bark, their graceful figures and bright-colored clothing forming picturesque groups in the forest glades, and the whole air being loaded with the scent of the spice. Cinnamon peeling begins in May, at the end of the rains, and lasts till November. The peeling simply consists in slitting the bark and cutting it across, so as to turn it back; it is then soaked to remove the outer rind, and rolled up into quills about three feet long, and it is then fit for exportation. Cinnamon contains volatile oil, tannin, a mucilage, vegeto-animal coloring matter, an acid and a woody fibre.

CINNAMON.

## Figures and Tables

**Figure f1:**